# Cost-effectiveness of the anti-fibrotics for the treatment of idiopathic pulmonary fibrosis in the United States

**DOI:** 10.1186/s12890-021-01811-0

**Published:** 2022-01-10

**Authors:** Timothy M. Dempsey, Viengneesee Thao, James P. Moriarty, Bijan J. Borah, Andrew H. Limper

**Affiliations:** 1 David Grant Medical Center, US Air Force, 101 Bodin Circle, Travis AFB, CA 94535 USA; 2 Robert D. and Patricia E. Kern Center for the Science of Health Care Delivery, Mayo Clinic, 200 1st St SW, Rochester, MN 55905 USA; 3 Department of Pulmonary and Critical Care Medicine, Mayo Clinic, 200 1st St SW, Rochester, MN 55905 USA; 4 Division of Health Care Delivery Research, Mayo Clinic, 200 1st St SW, Rochester, MN 55905 USA

**Keywords:** Idiopathic pulmonary fibrosis, Cost-effectiveness analysis, Pirfenidone, Nintedanib

## Abstract

**Background:**

The anti-fibrotic medications nintedanib and pirfenidone were approved in the United States for use in patients with idiopathic pulmonary fibrosis several years ago. While there is a growing body of evidence surrounding their clinical effectiveness, these medications are quite expensive and no prior cost-effectiveness analysis has been performed in the United States.

**Methods:**

A previously published Markov model performed in the United Kingdom was replicated using United States data to project the lifetime costs and health benefits of treating idiopathic pulmonary fibrosis with: (1) symptom management; (2) pirfenidone; or (3) nintedanib. For the cost-effectiveness analysis, strategies were ranked by increasing costs and then checked for dominating treatment strategies. Then an incremental cost-effectiveness ratio was calculated for the dominant therapy.

**Results:**

The anti-fibrotic medications were found to cost more than $110,000 per year compared to $12,291 annually for symptom management. While pirfenidone was slightly more expensive than nintedanib and provided the same amount of benefit, neither medication was found to be cost-effective in this U.S.-based analysis, with an average cost of $1.6 million to gain one additional quality-adjusted life year over symptom management.

**Conclusions:**

Though the anti-fibrotics remain the only effective treatment option for patients with idiopathic pulmonary fibrosis and the data surrounding their clinical effectiveness continues to grow, they are not considered cost-effective treatment strategies in the United States due to their high price.

## Background

Idiopathic pulmonary fibrosis (IPF) is a chronic fibrosing lung disease with a reported prevalence of 35 per 100,000 people and a high five-year mortality rate [1]. Because of the progressive and fatal nature of the disease, as well as the multitude of co-morbidities that accompany it, the overall health care utilization of patients with IPF has been shown to be quite high [2–4]. In one Medicare claims study, IPF patients were found to have a higher risk of hospitalization and increased total medical costs (by more than $10,000) compared to matched Medicare controls without IPF [4]. Another retrospective cohort analysis found that annual all-cause medical costs per patient with IPF was nearly $60,000 [5]

Most cost analyses of IPF were done prior to the introduction of the anti-fibrotic medications, pirfenidone and nintedanib, which were approved in 2014 after randomized controlled trials (RCTs) showed these medications were effective at slowing the decline in lung function in IPF patients [6, 7]. Since then, several studies have confirmed the clinical efficacy of these drugs, including one recent observational study demonstrating that patients may have lower mortality and hospitalization rates when compared to those not on treatment [8–10]. Despite these benefits and the lack of other effective treatment options, there are still concerns about anti-fibrotics, including their cost (more than $100,000 per year in the United States), which may add to the economic burden of patients with IPF [11].

To date, there has been no cost-effectiveness analysis (CEA) of the anti-fibrotic medications in the United States (US). While other countries have performed CEAs with mixed results, these results are not generalizable to the US health care system because of the large differences in the price of the medications. For example, in the United Kingdom (UK), the annual list price of pirfenidone is equivalent to $36,070.80 US dollars (USD) compared to an annual charge for both medications in the US of more than $100,000 [12, 13]. Similarly, in Belgium, the annual list price of nintedanib is around $28,910 USD [14]. Due to the growing body of clinical effectiveness data surrounding the anti-fibrotic medications coupled with their high price, determining whether they are cost-effective treatment options for patients with IPF is vital for patients, advocacy organizations, policymakers, and clinicians as such information can help inform drug pricing negotiation and facilitate improved access to the medications to patients who may most benefit. Thus, using a previously published Markov model of patients with IPF, we performed the first cost-effectiveness analysis of pirfenidone and nintedanib in the United States.

## Methods

### Markov model

We used a previously published Markov model (from the UK) that incorporates U.S.-specific data and project the lifetime costs and health benefits of treating IPF with: (1) symptom management; (2) pirfenidone; or (3) nintedanib [12]. Efficacy outcomes included mortality, lung function decline (as a surrogate for disease progression), and acute exacerbations. Outcomes were informed by three prior sets of randomized controlled trials (CAPACITY, TOMORROW, INPULSIS I, and INPULSIS II) and a network meta-analysis designed by the UK group [7, 12, 15, 16].

**Fig. 1:**
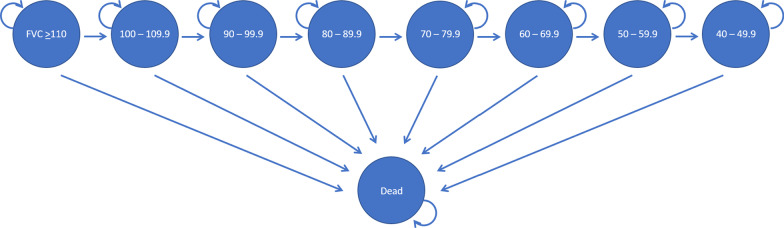
Markov model of lung function for idiopathic pulmonary fibrosis. The Markov model breaks up a disease into health states (in circles, i.e. 100% FVC, death) and health events. When a health event happens (e.g. worsening lung function), it is denoted as a transition (signified by arrows) to another health state. *FVC* forced vital capacity

A schematic of the Markov model is shown in Fig. 1. Individuals with IPF entered the Markov model with no history of exacerbation and treated with one of three options (symptom management, pirfenidone, or nintedanib). Every cycle (three months), individuals could experience an exacerbation (as defined by the 2016 International Working Group Report), a decrease in lung function, or death [17]. Exacerbations were tracked in the model using a tracker variable. Lung decline was permanent so that those who experienced a decline could not improve to health states with better lung function. Consistent with the UK model, death could occur in any health state or at the point that an individual’s lung function dropped below 40% forced vital capacity (FVC). Individuals were followed until death.

We assumed a health care payer’s perspective. Costs included direct medical costs for treatment, follow-up, and hospitalizations related to acute exacerbations. We did not include out-of-pocket costs or non-medical costs such as productivity loss, time off from work, or travel and childcare expenses incurred as a result of follow-up care.

### Model parameters

We used published survival curves from a network meta-analysis conducted on patient-level data from the TOMORROW and INPULSIS trials to inform baseline mortality [12]. The network meta- analysis also informed the risk of acute exacerbations which was set to 1.97% per cycle.

Like the UK model, we assumed that following an acute exacerbation, patients would experience an increased risk of death, set as an increased rate of 1.40 per cycle. Finally, the network meta-analysis also estimated the rate of lung function decline which varied by initial %FVC and was set between 3.2 and 8.9% per cycle.

The cost for pirfenidone and nintedanib were estimated from the OptumLabs Data Warehouse, which is a large de-identified administrative claims database comprised of commercially insured and Medicare Advantage individuals from across the U.S [18]. We pulled from the OptumLabs Data Warehouse the 2020 Bluebook costs per 801 mg of pirfenidone and 100 mg of nintedanib. We multiplied the costs/mg by 365 days for the annual costs.

The cost for follow-up and symptom management, was calculated by summing the 2020 CMS Fee Schedule reimbursement rate for the following procedure codes: 99,205 (pulmonary office visit); 71,250 (CT chest); 94,010, 94,013, 94,729 (pulmonary function tests); E0424, E0441, E0443 (home oxygen); 93,303–93,304 (Echocardiogram); 94,618 (6-min walk test); G0237–G0239, G0424 (pulmonary rehabilitation); 99,497–99,498 (palliative care); and 99,215 (primary office visit) [19]. We estimated that patients with IPF would need a: pulmonary office visit every 3–6 months; CT chest every 6–12 months; pulmonary function test every 3–6 months; 2–4L of oxygen therapy; 1 echocardiogram per year; 6–minute walk test every 6–12 months; and one round of pulmonary rehabilitation. Our clinical experts (TD and AL) selected the procedure codes to include in our costing model. We updated the average hospitalization cost of an acute exacerbation, estimated by Yu et al., to 2020 dollars using the U.S. Bureau of Labor Statistics’ Consumer Price Index for hospital services [20, 21].

The annual cost for the anti-fibrotic medications were $113,193 for pirfenidone and $112,357 for nintedanib. Annual follow-up costs included: $8,916 for oxygen therapy, $890 for a pulmonary office visit and primary care office visit, $680 for a pulmonary function test, $401 for an echocardiogram, $241 for a CT, $160 toward palliative care, $62 for pulmonary rehabilitation, and $51 for 6-min walk test. Thus, the annual cost of follow-up for IPF patients was approximately $12,291.

Quality of life (QOL) varied by lung function and whether an individual had experienced an acute exacerbation. Previously, authors analyzed patient-level data from the INPULSIS trial and used the EQ-5D to measure QOL by % forced vital capacity (FVC) (Table 1). In a separate analysis, authors also used the INPULSIS trial to measure how acute exacerbations impacted QOL. We used the findings from this study and assumed that exacerbations decreased QOL by 0.140 in the initial cycle that it is experienced and decreased QOL by 0.078 in subsequent months [7]

**Table 1:** Estimates of the various parameters (means) utilized in the Markov Model, comparing symptom management to pirfenidone and nintedanib

Variable description	Symptom management	Pirfenidone	Nintedanib
Probabilities—beta and log-normal distributions			
Lung function decline	0.032–0.089	OR = 0.55 (0.09)	OR = 0.54 (0.08)
Mortality	Varies [12]	OR = 0.69 (0.19)	OR = 0.70 (0.19)
Acute exacerbation	0.0197	OR = 1.10 (0.58)	OR = 0.56 (0.18)
Additional mortality following AE	OR = 1.40 (0.20)	OR = 1.40 (0.20)	OR = 1.40 (0.20)
Quality of life—beta distribution			
Lung function [37]			
90–110%	0.8380 (0.1782)	0.8380 (0.1782)	0.8380 (0.1782)
80–89.9%	0.8105 (0.2051)	0.8105 (0.2051)	0.8105 (0.2051)
70–79.9%	0.7800 (0.2244)	0.7800 (0.2244)	0.7800 (0.2244)
60–69.9%	0.7657 (0.2380)	0.7657 (0.2380)	0.7657 (0.2380)
50–59.9%	0.7387 (0.2317)	0.7387 (0.2317)	0.7387 (0.2317)
40–49.9%	0.6634 (0.2552)	0.6634 (0.2552)	0.6634 (0.2552)
Exacerbation, disutility [7]			
First cycle, post-exacerbation	− 0.140 (0.047)	− 0.140 (0.047)	− 0.140 (0.047)
Subsequent cycles	− 0.078 (0.032)	− 0.078 (0.032)	− 0.078 (0.032)
Costs (2020 U.S. dollars)—log-normal distribution			
Drug (annual) [18, 19]	–	$ 113,193	$ 112,357
Acute exacerbation, per episode [20]	$ 14,731 (4,026)	$ 14,731 (4,026)	$ 14,731 (4,026)
Follow-up care (annual) [19]	$ 12,291 (710)	$ 12,291 (710)	$ 12,291 (710)
Oxygen therapy	$ 8,916	$ 8,916	$ 8,916
Pulmonary office visit	$ 890	$ 890	$ 890
Primary care	$ 890	$ 890	$ 890
Pulmonary function test	$ 680	$ 680	$ 680
Echocardiogram	$ 401	$ 401	$ 401
CT chest	$ 241	$ 241	$ 241
Palliative care	$ 160	$ 160	$ 160
Pulmonary rehabilitation	$ 62	$ 62	$ 62
6-min walk test	$ 51	$ 51	$ 51

Lung function values reported are forced vital capacity. Costs are in United States dollars

*SD* standard deviation, *OR* odds ratio

### Markov simulation

Our model accounted for the costs, acute exacerbation rates, and mortality associated with treatment, as well as the impact that IPF had on lung function, and quality of life in the long term. All future costs and quality-adjusted life years (QALYs) were discounted at 3% per year [22]. The model was constructed and analyzed in TreeAge Pro v. 2019. We used the dampack package in R to plot results [23].

### Cost-effectiveness analysis

For the cost-effectiveness analysis, we followed the guidelines outlined by the *2nd Panel on Cost-Effectiveness in Health and Medicine* [22]. First, we ranked strategies by increasing costs. Then, we checked for dominated strategies (having higher costs and lower effectiveness than opposing strategies), which we eliminated. For the remaining strategies (those having higher costs and higher effectiveness than opposing strategies) we calculated the incremental cost-effectiveness ratio (ICER), defined as the additional cost of the next costly strategy divided by the additional QALYs gained. ICERs were compared to a willingness-to-pay (WTP) of $100,000 per QALY gain. Strategies with ICERs that were below the WTP threshold were considered cost-effective. ICERs above the WTP threshold were considered too costly and therefore not cost effective.

### Sensitivity analysis

In order to assess the uncertainty surrounding model parameters on the cost-effectiveness results, we performed threshold and probabilistic sensitivity analysis (PSA). In the threshold analysis we varied each parameter, one at a time while holding all others constant, to determine when the cost-effective strategy would change at a WTP of $100,000.

In the PSA, we repeated the cost-effectiveness analysis using 1,000 random samples of size 10,000 for which the underlying parameters were drawn from their estimated probability distribution (distributions are specified in Table 1). We constructed the cost-effective acceptability curve (CEAC) to display the proportion of times a strategy was cost-effective among the 10,000 samples. We also identified the cost-effective acceptability frontier (CEAF). The CEAF is different from the CEAC as it displays the strategy with the most expected benefit on average across all samples of the PSA at a specific WTP value [24].

## Results

### Cost-effectiveness analysis

Symptom management was associated with lifetime costs and benefits of $79,815 and 3.78 QALYs, respectively. Nintedanib was associated with lifetime costs and benefits of $675,544 and 4.15 QALYs, respectively. This amounts to an incremental cost-effectiveness ratio of $1.6 million per QALY (Table 2). Pirfenidone had lifetime costs of $688,778 and benefits of 4.10 QALYs.

**Table 2:** Cost, effect, and incremental cost-effectiveness ratio of symptom management, pirfenidone, and nintedanib for patients with idiopathic pulmonary fibrosis

	Cost ($)	Δ Cost	Effect (QALYs)	Δ Effect	ICER
Symptom management	$ 79,815		3.78		
Nintedanib	$ 675,544	$ 595,729	4.15	0.37	$ 1,601,224
Pirfenidone	$ 688,778	$ 13,233	4.10	− 0.05	*DOMINATED*

Cost in United States dollars. QALYs-quality-adjusted life years

*ICER* incremental cost-effectiveness ratio

### Sensitivity analysis

**Fig. 2:**
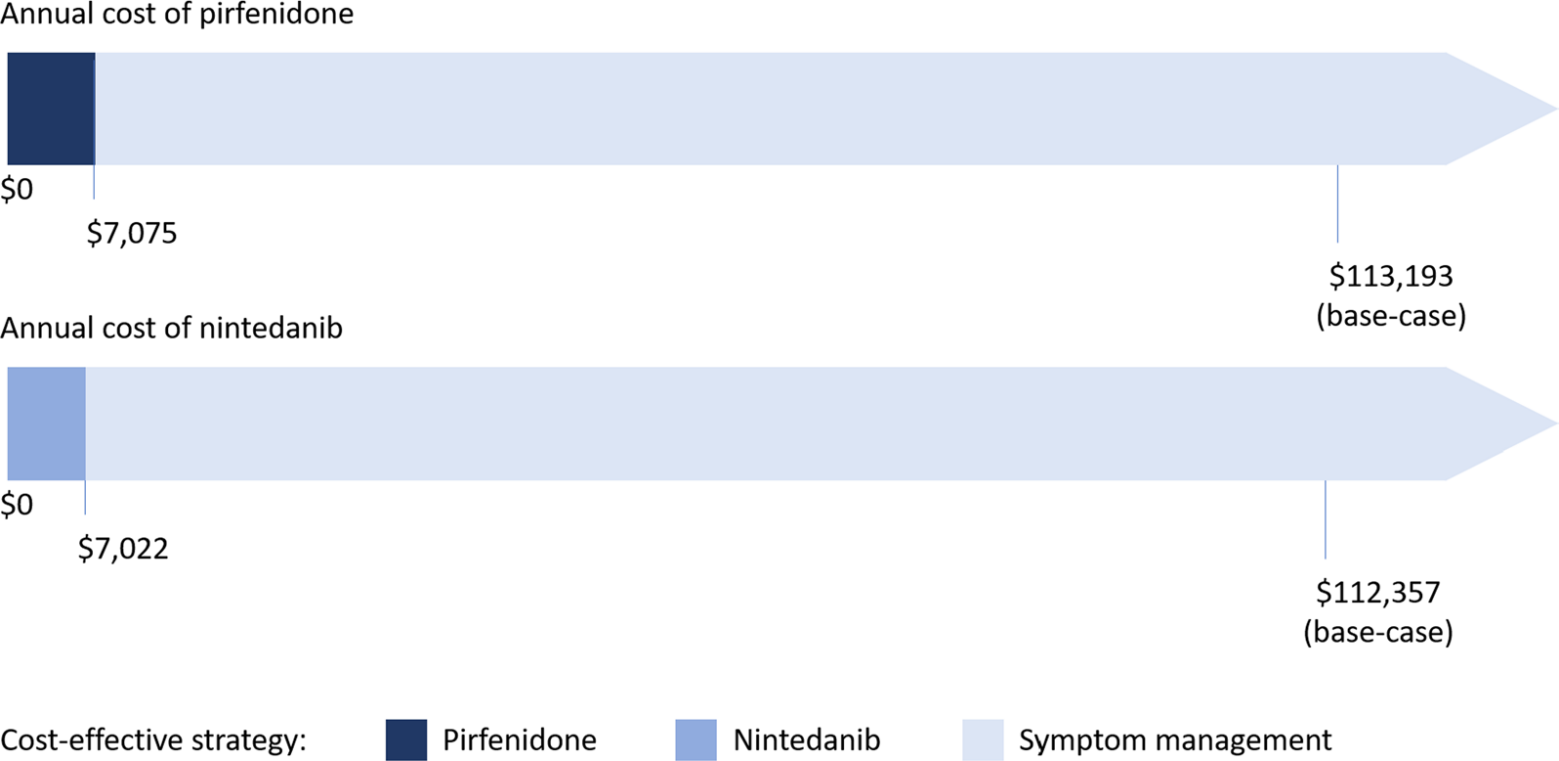
Schematic of threshold analysis demonstrating the price at which pirfenidone and nintedanib are cost-effective treatment strategies compared to symptom management

For threshold analyses, we set our WTP to $100,000 and evaluated two categories of variables: cost of treatment and treatment-related outcomes (Fig. 2). We found that pirfenidone could be cost-effective compared to symptom management and nintedanib if the annual cost of pirfenidone was between $0 and $7075. At pirfenidone cost values ≥ $7075, symptom management was the only cost-effective strategy for IPF. When the cost of nintedanib was between $0 and $7022 nintedanib was cost-effective compared to symptom management and pirfenidone. At cost values ≥ $7022, symptom management remained the only cost-effective strategy.

Pirfenidone and nintedanib were never cost-effective at current pricing when considering treatment-related outcomes, even with significant improvement in mortality, decreased rate of acute exacerbations, and decreased decline in lung function. For example, even if pirfenidone decreased mortality by 90% (base-case: 31% decrease) symptom management remained cost-effective (ICER: $238,420). Furthermore, if nintedanib decreased the rate of acute exacerbations by 90% (base-case: 44% decrease) symptom management remained cost-effective (ICER: $567,936).

**Fig. 3:**
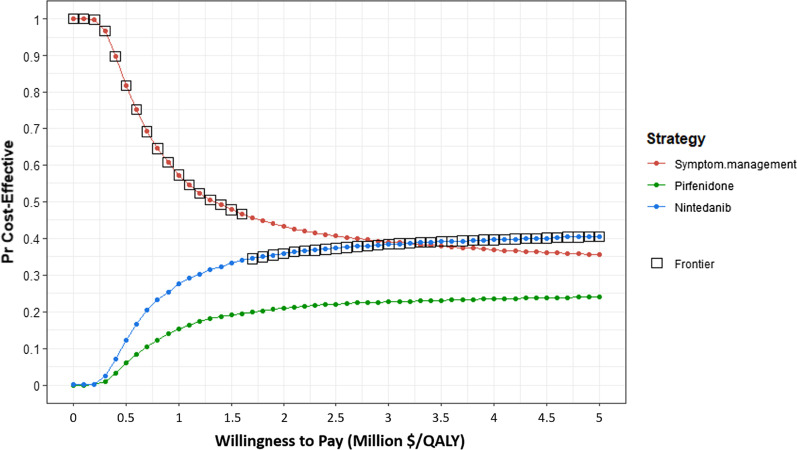
Probabilistic sensitivity analysis of symptom management (red) compared to pirfenidone (green) and nintedanib (blue). X-axis is our WTP. Y-axis is the proportion of times a specific treatment strategy was cost effective among 10,000 simulations

The PSA showed that among 10,000 simulations, symptom management was cost-effective ≥ 45% of the time at WTP thresholds between $0 and $1.6 million (Fig. 3). Although nintedanib was only cost-effective in 34% of 10,000 simulations at WTP of $1.6 million, nintedanib had higher expected benefits than symptom management and pirfenidone and was therefore cost-effective starting at $1.6 million (see CEAF, Fig. 3). Pirfenidone was never cost-effective compared to symptom management and nintedanib at WTP thresholds between $0 and $5 million.

## Discussion

This is the first cost-effectiveness analysis of the anti-fibrotic medications for patients with IPF in the United States since their approval in 2014. Using US data, we replicated a previously published Markov model from the United Kingdom based on clinical trial data and a network meta-analysis and found that, while nintedanib is somewhat more cost-effective than pirfenidone, overall the two medications are currently too expensive to be considered cost-effective treatment options for IPF [12].

Since their approval, observational studies and registry data from several countries have confirmed the anti-fibrotics real world effectiveness in slowing the decline in lung function in addition to suggesting a possible decrease in hospitalizations and mortality [10, 25, 26]. Though the clinical effects of the anti-fibrotics are becoming undeniable, the adoption of the medications has remained lower than expected, with registry and real world data suggesting somewhere between 26 and 70% of IPF patients are currently receiving prescriptions for the medications [13, 27–29]. One potential reason for this lower than expected adoption is their high price in the US, with out-of-pocket costs to patients of nearly $400 U.S. dollars per month for the medications and a total annual charge (out-of-pocket cost plus health plan payment) of more than $110,000 for each drug [13]. These prescription costs are in addition to the other health care expenses for patients with IPF, including their high rate of co-morbidities (and other prescription medications that go along with these), frequent need for oxygen supplementation, risk of hospitalizations, repeat office visits, imaging, and lab testing [3, 4, 26, 27].

Given the growing data on clinical effectiveness, the low adoption of the anti-fibrotics, and the high cost of both the medications as well as other health care costs for patients with IPF, determining the cost-effectiveness of both pirfenidone and nintedanib in the United States is essential. In this first US analysis, we found that, despite the clinical effectiveness of the medications, they are not close to being cost-effective at their current price. In our model, pirfenidone was slightly more expensive than nintedanib and provided the same amount of benefit; however, both medications’ ICER scores were far too high, with a net benefit of 0.37 QALY over symptom management. This translates into a staggering cost of $1.6 million USD to gain one additional QALY with nintedanib, a value 16 times higher than the commonly used willingness-to-pay threshold of $100,000 [30]. The main driver of the high ICER for the medications is the cost to obtain the medications, which is similar to the findings from the European CEA data [12, 14, 31]. As with an evaluation from the UK (which our Markov model was based on), the results are sensitive to changes from acute exacerbations, which is potentially the reason nintedanib had slightly more cost-effectiveness than pirfenidone given its ability to reduce acute exacerbations in prior studies [32]. However, even if the efficacy of the medications (including their ability to reduce acute exacerbations and death) is significantly increased as was done in our sensitivity analysis, the drugs are still not cost-effective at their current US price.

Though the results from prior European cost-effectiveness analyses of the anti-fibrotics are not directly applicable to the United States given the heterogeneity of the various health care systems as well as the large difference in the price of the medications (around $30,000 USD on average in Europe versus more than $110,000 in the US), these European studies also observed that the anti-fibrotic medications are not cost-effective, even with lower list prices [12, 14, 29]. One systematic review found that, of the ten studies published in Europe, none considered the anti-fibrotics to have an acceptable ICER, though the magnitude of the ICER was far higher in our study [33]. This evaluation also discovered that, in the majority of cases, nintedanib was the more cost-effective agent than pirfenidone. Despite the differences in payment models, prescription drug prices, overall health care costs, and insurance coverages between the US and Europe, the lack of cost-effectiveness of the anti-fibrotics is consistent.

While the findings of this CEA are intriguing, the analysis has several limitations. First, though the Markov model has been used in several prior publications, it was derived from trials and a network meta-analysis with several assumptions made in order to create the model. For a disease as complex as IPF, it can be difficult to make assumptions, which could certainly have impacted the results. Further evaluation based on real world data would be useful in helping corroborate these results. Second, administrative codes were used to evaluate the costs of the non-medical therapies and diagnostic testing for both the treatment and supportive care groups. These administrative codes may include some miscoding, which also could have altered our findings, including the cost for patients in both the treatment and supportive care group. Additionally, our model did not take into consideration combination treatment with both pirfenidone and nintedanib. Future models may wish to include this option given recent studies suggesting benefit to dual therapy [34–36]. We found that the cost of just one drug was already too expensive to be considered cost-effective (even with altering their current efficacy to demonstrate significantly improved outcomes); therefore, combination therapy is exceedingly unlikely to be cost-effective. Disease severity was also not a variable in our model, which should be evaluated in future studies given the possibility that this could impact cost-effectiveness. Finally, given the various different insurance models offered within the US health care system, the list prices for the medications and the different health care interactions may not accurately reflect the amount individual IPF patients pay for their care. Many pharmaceutical companies also offer prescription assistance programs that can offset the cost of expensive medications, including the anti-fibrotics, which also could have impacted our analysis. However, these programs are largely for uninsured and underinsured patients and so many patients with IPF will not be eligible. All of these limitations are important to consider; however, given how high we found the ICER of the anti-fibrotics to be, it is unlikely that they significantly change the main findings from this study.

## Conclusions

While these are the only drugs available to treat patients with IPF, their overall benefit as assessed through cost-effectiveness evaluation has not been conducted for the US setting. Our cost-effectiveness study attempted to fill this void by evaluating both clinical effectiveness and their corresponding costs through a single metric, ICER, and found that the anti-fibrotic medications are not cost effective treatment options in the United States. Our study will help inform policy makers including payers (Medicare and commercial insurance companies) while negotiating with the corresponding drug makers on potential price drops and prescription assistance programs. In addition, our study can also inform payers in structuring the coverage of these drugs in their formularies to facilitate improved access of the medications to patients that will benefit the most. For a disease as deadly and complex as IPF, having any available medical therapy for patients remains a major breakthrough; however, policy makers, patients, clinicians, and advocacy groups should continue to assess the value the medications provide clinically while also balancing their costs.

## Data Availability

The data from the model described in this study are previously published and publicly available in the articles cited in the manuscript. Other data is available from OptumLabs Data Warehouse but restrictions apply to the availability of these data, which were used under license for the current study, and so are not publicly available. Data are however available from the authors upon reasonable request and with permission of OptumLabs Data Warehouse.

## Abbreviations

CEA: Cost-effectiveness analysis
CEAC: Cost-effective acceptability curve
CEAF: Cost-effective acceptability frontier
FVC: Forced vital capacity
ICER: Incremental cost-effectiveness ratio
IPF: Idiopathic pulmonary fibrosis
PSA: Probabilistic sensitivity analysis
QALY: Quality-adjusted life years
QOL: Quality of life
RCTs: Randomized controlled trials
UK: United Kingdom
US: United States
USD: United States Dollars
WTP: Willingness-to-pay
